# A Conceptual Framework to Integrate Research Programs on Self-Regulation in Education

**DOI:** 10.1007/s10648-026-10196-7

**Published:** 2026-08-10

**Authors:** Franz Wortha, Peter Gerjets, Roger Azevedo, Matthew Bernacki, Birgit Brucker, Babette Bühler, Ann-Christine Ehlis, Enkelejda Kasneci, Akira Miyake, Kou Murayama, Benjamin Nagengast, Brent Roberts, Maike Tibus, Ulrich Trautwein

**Affiliations:** 1 Department of Mathematics Education, Loughborough University, Loughborough, UK; 2 LEAD Graduate School and Research Network, University of Tübingen, Tübingen, Germany; 3 Leibniz-Institut für Wissensmedien, Tübingen, Germany; 4 School of Modeling, Simulation and Training, University of Central Florida, Orlando, FL USA; 5 School of Education, University of North Carolina at Chapel Hill, Chapel Hill, NC USA; 6 Department of Education, Korea University, Seoul, Korea; 7 Technical University of Munich, Munich, Germany; 8 Department of Psychiatry and Psychotherapy, University Hospital Tübingen, Tübingen, Germany; 9 Department of Psychology and Neuroscience, University of Colorado Boulder, Boulder, CO USA; 10 Hector Research Institute of Education Sciences and Psychology, University of Tübingen, Tübingen, Germany; 11 Department of Psychology, University of Illinois, Urbana-Champaign, Urbana-Champaign, IL USA

**Keywords:** Self-regulation, Executive functions, Personality, Motivation, Self-regulated learning

## Abstract

Self-regulation (SR) has emerged as a pivotal construct in educational research, yet its diverse theoretical accounts often lack comprehensive integration. This paper addresses this by proposing an integrative conceptual framework that consolidates four core research traditions on SR in educational settings, each emphasizing distinct underlying mechanisms: (1) Stable Dispositions (e.g., personality traits; Matthews et al., 2009; Roberts et al., 2007; Song et al., 2020), (2) Limited Resources (e.g., working memory capacity and executive functioning; Friedman & Miyake, 2017; Paas et al., 2003; Sweller, 1994), (3) Driving Forces (e.g., motivation, interest, and affect; Eccles & Wigfield, 2002; Hidi & Renninger, 2006; Trautwein et al., 2019), and (4) Learning Activities (e.g., self-regulated learning, cognitive and metacognitive strategies; Winne & Hadwin, 1998; 2008; Zimmerman, 2000). By synthesizing these perspectives, our framework positions SR as a dynamic, interdependent system, emphasizing how interactions and compensatory mechanisms explain individual differences and influence learning outcomes. We further argue for an integrative methodological toolkit, leveraging advanced machine learning and computational models, to capture the multi-level, temporal, and social dynamics inherent in SR. This holistic approach is crucial for advancing a comprehensive and ecologically valid understanding of SR in educational contexts.

## Self-Regulation in Education

In the context of 21st-century education, self-regulation (SR) is increasingly recognized as a critical competency that complements domain-specific knowledge and skills (Greene et al., 2024; Inzlicht et al., 2021). It enables learners to effectively navigate current and future educational challenges (Dumont et al., 2010; National Research Council, 2011; Wolters, 2010). Broadly defined, SR refers to the capacity to monitor and manage one’s cognitions, emotions, motivations, and behaviors in pursuit of personal goals or in response to contextual demands (e.g., Dinsmore et al., 2008; Roebers, 2017). This capacity is widely regarded as both a key determinant and a central outcome of educational processes, making it a subject of significant interest to both researchers and practitioners (Moore et al., 2015; Pintrich, 2000; Zimmerman, 2000).

Reflecting its central importance, a substantial body of research has examined various dimensions of SR in educational contexts. This includes investigations into its antecedents, underlying mechanisms, targeted interventions, and its relationship with important educational outcomes such as academic achievement and student well-being (Duckworth & Seligman, 2005; Schunk & Greene, 2018b). Despite this extensive scholarship, the field remains highly fragmented. For example, investigations under the umbrella term of SR in education range from studies of the cognitive foundations of SR (e.g., executive functioning), to the regulation of cognitive and metacognitive activities during learning (e.g., the core of self-regulated learning, SRL), to analyses of how effort, engagement, and perseverance can be supported during learning (e.g., motivational processes), and how stable dispositions shape educational careers (e.g., personality). The lack of a cohesive conceptual framework has impeded cumulative progress and contributed to theoretical ambiguity (e.g., Alexander, 2008; Dinsmore et al., 2008; Kaplan, 2008; Lajoie, 2008; Schunk, 2008).

One major source of fragmentation stems from the jingle-jangle fallacies, which describe key conceptual confusions in psychological research (e.g., Wulff & Mata, 2026). Specifically, a jingle fallacy occurs when the same label (the same “jingle”) is used to refer to distinct constructs (measured by different indicators), so that the same label represents separate phenomena. A jangle fallacy, on the other hand, occurs when a core construct is referred to with different labels (resulting in a “jangle”), even though overlapping indicators are used for measurement. In particular, the introduction of novel labels for constructs that conceptually overlap with existing ones (a jangle fallacy) induces confusion for those who wish to study psychological processes, complicates theory development, and undermines efforts to synthesize findings across studies (Elson et al., 2023). Naming different things with identical names (a jingle fallacy), on the other hand, is often easier to handle because it simply requires differentiating a heterogeneous construct into more precise subconstructs (e.g., different types of inhibition, different types of engagement). Examples of jingle-jangle fallacies are frequent in educational psychology, and they can stymie research and theory development and misdirect the energy of educational practitioners (Hanfstingl et al., 2024; Teyssier-Roberge et al., 2025).

The terms SR and SRL themselves illustrate a potential jingle fallacy, which was one of the central motivations for the present paper: the label “self-regulation” has increasingly been applied across disciplines to partially overlapping yet distinct constructs and processes. However, its application lacks uniformity, encompassing phenomena that vary significantly in scope and temporal scale. The much narrower term SRL, in comparison, has a relatively clear conceptual core according to reviews of SRL models (Panadero, 2017; Puustinen & Pulkkinen, 2001), centering on cyclical processes of planning, executing, monitoring, and adapting cognitive and behavioral learning activities (or learning strategies composed of such activities). Importantly, these models also emphasize that effective regulation requires standards against which current progress and outcomes are evaluated (the broader nature, origin, and effects of these standards are, of course, a research topic of their own in other fields of research related to SR, such as motivation). Nevertheless, as these reviews also show, all SRL models differ greatly in their specific assumptions surrounding this conceptual core. These assumptions might be about different types of information processing, motivational processing, environmental control, emotion regulation, or volitional control. Therefore, adopting a specific model of SRL implies a specific focus of attention, using specific indicators to measure SRL. As a result, different SRL models lead researchers to view identical learning episodes in a different light. This can lead to a research landscape where it becomes unclear whether the different components integrated into SRL models, such as metacognition, motivation, volition, or emotions, should really be considered components of a monolithic SR(L) construct that is, however, conceptualized very heterogeneously in different models (i.e., a jingle fallacy). Alternatively, one might reserve the term SRL for the core cycle of planning, executing, monitoring, and adapting learning activities that is shared by all theoretical models (avoiding a jingle fallacy). In this case, SRL models would describe different nomological contexts of SRL, i.e., different sets of variables that influence the SRL learning cycle or are influenced by it. This narrower conception of SRL might be preferable because it avoids the jingle fallacy and does not blur the lines between various, potentially distinct constructs. Beyond SR and SRL themselves, several jingle fallacies have been discussed in the context of SR. For instance, self-control has been used to describe both the narrow cognitive process of impulse inhibition (Hofmann et al., 2012) and a broad, domain-general trait heavily overlapping with conscientiousness (Tangney et al., 2004). Other jingle fallacies related to SR in education include the label executive functioning (EF) being used for various constructs. Whereas contemporary models of EF focus on a set of cognitive components (working memory, inhibition, etc.; Miyake et al., 2000), other models of SRL conceptualize them more broadly (e.g., task analysis, strategy monitoring, and strategy control; Borkowski et al., 2000) with strong overlap to metacognition (thereby resulting in a jangle fallacy of describing similar things with different labels at the same time). Accordingly, reviews of EF and metacognition highlighted that such conceptual confusion is persistent in research and that integrative approaches are required (Dinsmore et al., 2008; Kim et al., 2023; Roebers, 2017). Jingle fallacies in education further propagate when new umbrella terminology is invented to inform more general audiences of fields of theory and research that could be incorporated into efforts to address educational problems in practice (Teyssier-Roberge et al., 2025). For example, terms such as “non-cognitive factors” or “21st-century skills” commonly appear in general educational outlets for a broader public and induce a jingle fallacy because a set of varying processes that are not cognitive (including the metacognitive, motivational, and affective) or a broad set of varying “soft skills” to be developed in students are subsumed under a single label, mistakenly suggesting that these are precisely defined sets of skills for students (Gutman & Schoon, 2013) and workers (Kautz et al., 2017).

Whereas jingle fallacies (misleadingly) suggest that identical labels denote identical things, the opposite is true for jangle fallacies, where different labels suggest different things. Grit represents a high-profile candidate for a jangle fallacy, where grit was defined as sustained passion and perseverance for long-term goals (Duckworth et al., 2007). Studies of grit revealed conceptual overlap with and reliance on measurement approaches that were highly similar to existing theoretical constructs with substantial research bases, including conscientiousness, a broader trait that encompasses responsibility and organization (Costa & McCrae, 1992), and motivational constructs (Muenks et al., 2018), which capture the majority of the variance that grit could explain in academic achievement. However, they differ in terms of their theoretical underpinnings and temporal levels of analysis, leading to divergent implications for understanding SR processes and predicting academic outcomes (Credé et al., 2017). Other educational examples of jangle fallacies include the constructs of (academic) self-concept and self-efficacy, which have been shown to be so closely related that they are indistinguishable empirically (Marsh et al., 2019). These fallacies can be further exacerbated by measurement issues. For instance, it has been shown that different measures of self-efficacy, such as academic and test-related self-efficacy, are distinct empirically, thereby creating a full jingle-jangle fallacy encompassing both types of problems (Marsh et al., 2019). Similarly, studies have shown that EF measures based on self-reports are empirically distinct from measures based on experimental paradigms because they reflect personality facets (e.g., conscientiousness or neuroticism), which is not the case for experimental measures (Buchanan, 2016; Eisenberg et al., 2019). Such issues are becoming increasingly prevalent across all fields of psychological research, and a proliferation of measures has been well documented (Anvari et al., 2025; Elson et al., 2023; Wulff & Mata, 2026). For example, Anvari et al. (2025) estimated that in educational and developmental psychology, the cumulative number of constructs and measures has more than doubled over the last decade, highlighting the pressing need for an integrative approach that can reconcile such conceptual divergences and overlaps.

To address this issue, in the present paper we adopt a Lakatosian (1970; 1978) framework of scientific research programs to scope and examine the landscape of SR theory in education. From this perspective, different research traditions can be understood as families of research programs that are structured around a common *hard core* of foundational assertions, which is protected from immediate falsification by a *protective belt* of auxiliary hypotheses, methods, and operational constructs. This approach allows us to treat each tradition as a distinct, yet rationally comparable, set of research programs where theoretical progress is measured by a program’s long-term capacity to generate novel, testable predictions rather than by immediate conceptual agreement among researchers. For instance, we will use the label *Learning Activities* to describe the *hard core* of a research tradition analyzing SRL with a focus on metacognitive monitoring and control of learning activities as central mechanisms facilitating strategic learning (Winne & Hadwin, 1998). Although, for instance, the importance of basic cognitive functioning underlying monitoring and control, such as EF and working memory, is acknowledged in this research tradition, it does not represent its foundational assumptions and mechanisms (Dörrenbächer-Ulrich & Bregulla, 2024). In contrast, we will use the label *Limited Resources* to refer to the *hard core* of a research tradition in SR that foregrounds basic cognitive capacities, such as working-memory capacity or EF, as fundamental enablers of behavioral regulation (Hasher & Zacks, 1988; Miyake et al., 2000). This tradition has also studied the role of these cognitive capacities for learning, but the monitoring and control of Learning Activities does not represent a central theoretical concern in this research field. These distinct theoretical cores often share auxiliary constructs from other research traditions, such as the amount of invested mental effort (Salomon, 1983) or goal-focus (Ames, 1992; Dweck, 2017), which function within different conceptual ecosystems, further complicating integration across traditions.

**Fig. 1:**
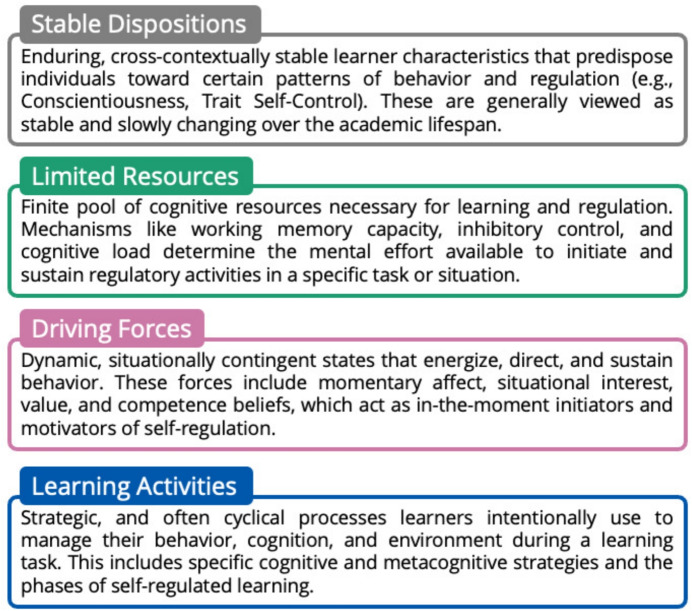
The four research traditions included in the framework on SR in education. They are depicted in order of the subsequent sections

This paper seeks to address the central question: How can divergent conceptual perspectives on SR be integrated to form a more unified and comprehensive understanding of SR in educational settings? To this end, we categorize existing research programs into four broad traditions, each of which is characterized by its core explanatory construct family: (1) Stable Dispositions, (2) Limited Resources, (3) Driving Forces, and (4) Learning Activities (see Fig. 1). These traditions differ in several key respects, including their assumptions about the stability and malleability of SR processes, their preferred methodological approaches, and their (temporal) levels of analysis. For example, whereas the Stable Dispositions and Limited Resources traditions tend to emphasize relatively stable traits or capacities, the Driving Forces and Learning Activities traditions focus on dynamic, situationally contingent processes and states. Methodologically, these programs range from trait-based psychometric assessments to fine-grained, process-oriented studies using think-aloud protocols or trace data (Greene & Azevedo, 2009). Regarding (temporal) granularity, they span multiple levels of analysis, from basic neurocognitive processes underlying EF (Diamond, 2013) to longitudinal patterns of strategic learning behavior (Zimmerman, 2000) and lifelong development of personality traits such as conscientiousness (Spielmann et al., 2022). Building upon a complementary authorship team that represents the distinct positions of the four research programs, we aim to decompose the phenomena invoked in models of SR in education from different theoretical and methodological perspectives. In doing so, we follow a more encompassing approach than previous integrative work on SR and related constructs (EF, metacognitive monitoring; e.g., Roebers 2017, Theobald et al. 2026) by incorporating a broader range of theoretical perspectives on SR in education.

A key dimension of integration involves recognizing that learners’ states, traits, and processes can very often function both as the subject and the object of SR (Efklides, 2011). For instance, in SRL, metacognitive processes (as the subject) are deployed to regulate cognitive strategies (as the object), which can themselves be used (as a subject) to regulate specific Learning Activities (as an object). Similarly, within the Limited Resources tradition, EFs such as inhibition, shifting, and updating (Miyake et al., 2000) can be understood, on the one hand, as basic mechanisms (the subject) enabling the regulation of attention and behavior (the object). On the other hand, however, we can also deploy attentional and behavioral choices to regulate the short-term availability or necessity of executive resources.

By systematically examining the theoretical assumptions, mechanisms, and constructs associated with each of these four traditions, this paper aims to develop a conceptual framework that facilitates the integration of their insights. In doing so, we seek to advance a more nuanced and comprehensive model of SR in education, one that acknowledges its multifaceted nature and its central role in promoting learner success across diverse academic contexts and trajectories. To this end, in the following section, the four identified research traditions are introduced (see Fig. 1) before their integration into a framework of SR in education is discussed.

The four research traditions investigated in the following section were selected because they present distinct assumptions, mechanisms, and constructs of individual-level SR in education. Whereas the following sections focus on the four identified traditions and their integration, we acknowledge that this represents a necessary limitation in scope. Factors such as social context and co-regulation (e.g., Järvelä et al. 2018), environmental influence, gender, and socioeconomic status (e.g., Cuartas et al. 2022, Matthews et al. 2009) are undeniably vital determinants of SR in education. However, based on our selection criteria, we argue that these are best classified as antecedent or contextual variables that influence the state or development of the four core internal mechanisms, not as mechanisms of SR themselves. Therefore, to maintain theoretical coherence and focus on the individual learner’s internal system, we limit the scope of the framework to these four traditions. Potential impacts on the learner through social, environmental, and contextual factors are discussed in the final section of this paper.

### Stable Dispositions

While the manifestation of SR in learning is often dynamic and context-dependent, influenced by immediate task demands and environmental cues, the capacity for and tendencies toward SR are also rooted in more enduring personal characteristics of the learner. SR research programs centered around Stable Dispositions focus on these relatively consistent patterns of thoughts, feelings, and behaviors that provide a foundational layer, influencing how individuals approach and engage in self-regulatory processes across various learning situations and over time. In contrast to more malleable aspects of SR, such as context-specific Learning Activities or motivational states (e.g., self-efficacy for a specific class), these dispositions offer a more distal perspective on the underlying factors that shape self-regulatory tendencies.

Prominent examples of these Stable Dispositions include personality traits such as conscientiousness (Costa & McCrae, 1998, 2008), as well as stable social-cognitive and motivational constructs such as academic self-concept, dispositional interest (Hidi & Renninger, 2006; Marsh, 1990; Rieger et al., 2017), vocational interests (Gati, 1991; Holland, 1997; Low et al., 2005; Nye et al., 2012), mindsets (Yeager & Dweck, 2012), or even cognitive abilities (e.g., Roberts et al. 2007). For instance, a person’s cognitive processing speed is a prominent cognitive trait that has been postulated to be a foundation of individual differences in general cognitive abilities (Mashburn et al., 2024). Further, research has shown that facets of achievement goals (Scherrer et al., 2024), vocational interests (Low et al., 2005), and individual interest (Nagengast et al., 2011) demonstrate substantial temporal stability, underscoring their trait-like nature. Although trait constructs are characterized by their long-term stability, it is important to acknowledge their development and potential for change across the lifespan, particularly during childhood and adolescence (Bleidorn et al., 2022; Roberts et al., 2006), and even in later life (Roberts & Mroczek, 2008). Research in educational contexts has highlighted the significant role of dispositions such as conscientiousness (Costa & McCrae, 1998), grit (Duckworth et al., 2007), and stable motivational orientations (Trautwein et al., 2015) as robust predictors of academic success, often operating through their influence on academic effort and with a degree of independence from cognitive ability (De Raad & Schouwenburg, 1996; Poropat, 2009; Rimfeld et al., 2016).

In conclusion, the *hard core* of the Stable Dispositions research tradition assumes that SR is anchored in relatively enduring personal characteristics, such as personality traits, that shape a learner’s general approach to learning across contexts and time. This tradition posits that SR is a macro-level expression of a learner’s habitual regulatory profile, providing a consistent baseline for how an individual initiates and sustains effortful study behaviors. Unlike state-like regulatory events, these SR-related traits are typically viewed as stable and are assessed via self-reports or observer ratings rather than repeated, in situ measurements, as they are considered to be enduring rather than fleeting (Horstmann & Ziegler, 2020).

From a Lakatosian perspective, the *hard core* of this tradition identifies the trait-like personal constraints that shape how more proximal self-regulatory processes may unfold in specific learning situations. Whereas the *hard core* focuses on the relative stability of the individual, its *protective belt* connects these traits to dynamic behaviors and educational outcomes. Recent integrative work suggests that Stable Dispositions are closely linked to other self-regulatory traditions, particularly Driving Forces (e.g., motivational processes) and Learning Activities (e.g., learning strategies and SRL processes). Longitudinal and meta-analytic research has connected personality characteristics with motivation, academic achievement, and, to a lesser extent, learning strategies and self-regulatory behaviors (Lavrijsen et al., 2022; Nauzeer & Jaunky, 2021; Richardson et al., 2012). These findings indicate that dispositional characteristics shape how learners engage with and sustain self-regulatory processes across educational contexts.

For instance, conscientiousness has been linked to higher levels of Driving Forces (e.g., motivation), more frequent use of Learning Activities (e.g., SRL strategies), and better academic outcomes (Chamorro-Premuzic & Furnham, 2008; Eilam et al., 2009). Similarly, conscientiousness and openness have been associated with greater use of metacognitive and elaborative learning strategies (Chamorro-Premuzic & Furnham, 2008), whereas neuroticism often shows negative relationships with achievement, potentially due to emotional instability (Bidjerano & Dai, 2007). Interestingly, research further suggests that learners with strong trait-level self-regulatory characteristics may sometimes avoid situations that require extensive moment-to-moment regulation (Hill et al., 2014; Hofmann et al., 2012; Inzlicht & Roberts, 2024). This aligns with findings indicating that state self-control processes do not necessarily constitute the primary mechanism underlying individual differences in trait self-control (Inzlicht & Roberts, 2024).

### Limited Resources

Self-regulatory processes in educational contexts are not only shaped by stable learner characteristics but also fundamentally constrained by the availability and efficient utilization of limited cognitive processing resources. The regulation of cognition, behavior, motivation, and affect requires considerable mental effort and attentional control, relying heavily on resources such as working-memory capacity and executive functions (Hofmann et al., 2012; Miyake et al., 2000; Pintrich & Zusho, 2002).

Research on EF, much like that on SR, has evolved across various perspectives, leading to diverse definitions (Suchy, 2009). These range from models emphasizing core functions such as the ability to control attention (executive attention, Mashburn et al. 2024) or the updating, inhibition, and shifting of information in working memory (Miyake et al., 2000), later integrated into a hierarchical structure with a common EF factor (Miyake & Friedman, 2012), to distinctions between metacognitive (’cool’) and emotional (’hot’) EFs (Ardila, 2008; Zelazo & Carlson, 2012). A widely accepted definition describes EF as domain-general, higher-level cognitive processes that enable individuals to regulate their thoughts and actions toward goal-directed behaviors by influencing lower-level cognitive functions (Friedman & Miyake, 2017; Miyake et al., 2000). In essence, EF provides the cognitive foundation to represent goals and strategies and choose between them (Miller & Cohen, 2001), a cornerstone of goal-directed behavior inherent in SR. Furthermore, EF and working memory underpin cognitive control and metacognition (Alloway & Alloway, 2010; Cowan, 2014; Miyake et al., 2000), which are central to many self-regulatory activities. Beyond these, attentional and volitional resources, crucial for effort investment, emotion regulation, and impulse control, also play a role in successful goal achievement (Bauer & Baumeister, 2011; Gross, 2013; Mischel et al., 2011).

For clarification, when we introduce *Limited Resources* as an explanatory core concept in our framework, we do not refer to a primarily psychometric perspective on stable individual differences, as discussed in the section on Stable Dispositions. Instead, we emphasize the fluctuating availability of cognitive resources within a specific learning situation and the extent to which these resources can be allocated to ongoing self-regulatory processes. From this perspective, cognitive resource limitations are conceptualized as dynamic state components that vary across time and situations (e.g., due to fatigue or mind-wandering; Bühler et al., 2024b; Pessiglione et al., 2025). For instance, research has shown that working-memory capacity exhibits meaningful intraindividual fluctuations when assessed repeatedly across days or situations rather than only once as a stable trait variable (Dirk & Schmiedek, 2016). These fluctuations may themselves be influenced by factors such as bodily activity (Ruiz et al., 2025), sleep quality (Könen et al., 2015), and related situational conditions. Beyond these fluctuations, an additional issue concerns the extent to which currently available resources are occupied by competing demands rather than invested in task-relevant regulatory processes. For example, the working-memory resources of math-anxious learners may become consumed by worrisome thoughts and ruminations during problem solving, thereby impairing performance and learning (Szczygieł, 2021). Similarly, learners frequently devote substantial portions of their attentional resources to task-unrelated thoughts and mind-wandering rather than to ongoing regulatory demands within the learning situation (Bühler et al., 2024b). Consequently, when discussing *Limited Resources* as constraints on SR in educational contexts, we primarily refer to the fluctuating state of cognitive resources available and allocated within a given situation rather than to fixed trait-like capacities alone.

Interestingly, theories centered on Learning Activities have only selectively incorporated constructs associated with Limited Resources and rarely engage with the broader literature on cognitive resources and their fluctuations comprehensively (Pintrich, 2000; Winne & Hadwin, 1998). Some models acknowledge the importance of executive control processes for task analysis, strategy selection, and monitoring (e.g., Borkowski et al. 2000, drawing on Butterfield et al. 1995), thereby linking Learning Activities with aspects of EF research. However, these incorporations typically conceptualize cognitive resources from the perspective of regulating learning activities rather than as part of a broader research tradition concerned with the availability, allocation, and fluctuation of cognitive resources across contexts.

From the perspective of the Limited Resources tradition, executive functions and working-memory resources represent foundational cognitive mechanisms that enable or constrain regulatory behavior more generally. Metacognitive monitoring and control may therefore depend partly on the availability and efficient coordination of these underlying cognitive resources, particularly under conditions of high cognitive load, fatigue, stress, or distraction.

Nevertheless, recent integrative reviews indicate growing empirical convergence between research on executive functions and SRL processes. For example, Dörrenbächer-Ulrich and Bregulla (2024) concluded that executive functions, particularly working memory, inhibition, and cognitive flexibility, are consistently associated with core SRL activities such as planning, monitoring, and strategy adaptation. At the same time, that review highlighted substantial heterogeneity in conceptualizations and measurement approaches, emphasizing the need for a more theory-guided and process-oriented integration of cognitive resources into models of SR in education.

This relative lack of explicit integration within SRL theory stands in contrast to substantial empirical evidence highlighting the importance of cognitive resources for learning and academic achievement across various educational contexts (Alloway, 2006; Alloway & Alloway, 2010; Cirino & Willcutt, 2017; Diamond, 2013; Hutchins et al., 2013; Paas & Sweller, 2012; Xie et al., 2017). Robust relationships among working memory, EF, and academic success have been consistently demonstrated (Alloway & Alloway, 2010; Cirino & Willcutt, 2017; Diamond, 2013). Moreover, research in learning and instruction, particularly through the lens of Cognitive Load Theory (e.g., Paas and Sweller 2012), emphasizes the critical role of working-memory capacity in learning effectiveness, suggesting that optimal learning occurs when cognitive load is appropriately managed (Castro-Alonso et al., 2021), a notion supported by meta-analytic findings (e.g., Hutchins et al. 2013, Xie et al. 2017).

In conclusion, the *hard core* of the Limited Resources research tradition underscores the fundamental role of cognitive capacities, such as working memory and EF, as the underlying cognitive resources enabling SR in education. This tradition posits that SR is constrained by a finite pool of cognitive resources, where success is determined by the immediate availability and management of these physiological and cognitive capacities. Although these capacities are not always explicitly integrated into the core of SRL theories, the empirical evidence strongly supports the notion that these resources are indispensable for the effective deployment of self-regulatory strategies and ultimately influence learning and academic achievement.

From a Lakatosian perspective, the *hard core* of this tradition identifies the physiological and cognitive boundaries of regulation, whereas its *protective belt* provides the necessary support for, or constraints upon, the unfolding of other SR facets. Emerging integrative reviews have increasingly linked Limited Resources, particularly executive functions, with Learning Activities (e.g., SRL processes) and Driving Forces (e.g., motivational dynamics). For example, reviews by Dörrenbächer-Ulrich and Bregulla (2024) and Pretorius and Heyns (2026) highlight consistent associations among executive functions, metacognitive regulation, motivational processes, and academic functioning, suggesting that cognitive resources both support and constrain the deployment of self-regulatory activities across learning contexts. This growing integration is further reflected in efforts to bridge Cognitive Load Theory and SRL research (De Bruin & van Merriënboer, 2017), as well as in studies linking executive functions, metacognitive processes, and academic achievement (Effeney et al., 2013; Follmer & Sperling, 2016; Rutherford et al., 2018). In particular, executive-function components such as shifting and inhibition have been associated with both Learning Activities (e.g., SRL processes) and educational outcomes (Follmer & Sperling, 2016; Rutherford et al., 2018). Collectively, these findings suggest a dual role for cognitive resources within the broader framework: they provide the cognitive foundation necessary for self-regulatory processes while simultaneously constraining the extent to which other aspects of SR, such as Learning Activities, can unfold (Pintrich & Zusho, 2002).

### Driving Forces

The initiation and sustained engagement in learning activities and the corresponding regulation of thoughts and behaviors are fundamentally propelled by *Driving Forces* that energize these processes in specific directions (i.e., goals). These forces extend beyond mere cognitive capacity, as even the most capable learners may not achieve desired outcomes without the necessary motivation to utilize their skills (Zimmerman, 2000). Research analyzing these forces that drive learning encompasses a range of constructs, including situation-specific value beliefs and expectancies (Eccles & Wigfield, 2002; Nagengast et al., 2011), situational interest (Hidi & Renninger, 2006), types of achievement goals (Elliot & McGregor, 2001), and different achievement emotions along with their regulation (Harley et al., 2019; Pekrun, 2006). Each of these constructs has independently demonstrated links to learning and academic achievement. For instance, meta-analyses have shown a substantial positive impact of motivation (e.g., expectancy and value; Eccles and Wigfield, 2002) and motivational interventions on academic outcomes (Kriegbaum et al., 2018; Lazowski & Hulleman, 2016). Similarly, research reviews have highlighted the significant influence of emotions, particularly negative ones like anxiety and boredom, on learning processes (Loderer et al., 2020; Namkung et al., 2019; Tze et al., 2016). Furthermore, the interconnectedness of various Driving Forces within educational settings has been well-established (e.g., self-efficacy and interest; Huang, 2011, 2016; Rottinghaus et al., 2003).

It should be noted that, as in our treatment of Limited Resources as states in the current framework, we also conceptualize the Driving Forces experienced in a learning situation as transient states that differ structurally from the more general and stable motivational dispositions mentioned above (Vallerand, 1997). This is because we consider motivational and emotional forces to dynamically unfold in situ within a specific learning context during the process of setting and pursuing specific goal intentions. To clarify this point, consider the difference between personal interest as a stable disposition and situational interest as a driving force that develops in a specific learning context. According to Hidi and Renninger’s (2006) four-phase model of interest development (Renninger & Hidi, 2022), interest is understood as progressing over time from externally triggered situational interest, to maintained situational interest, to emerging (less-developed) individual interest, and finally to well-developed individual interest. This framework describes how an externally triggered, situational interest can gradually transform into a stable, trait-like personal interest through repeated positive engagement with content or activities. Situational interest is conceptualized as a psychological state characterized by increased attention, effort, and affect experienced in a particular moment. Personal interest, on the contrary, is defined as a person’s preference for one activity over others that is developed over time through a person’s constant and consistent interaction with that activity (possibly through rewarding processes; see Murayama et al. 2019; Murayama 2022). It represents an enduring predisposition to re-engage with a particular object or topic over time. When a person with an individual interest in a topic enters a situation that is related to this topic, they will experience an enhanced situational interest response and an enhanced engagement and processing experience. It is this situational response that we are focusing on when we discuss Driving Forces in a learning situation, rather than the enduring preference pattern that the person carries across time and that leads to the enhanced situational response.

This emphasis on dynamic, in-the-moment motivational and affective processes positions the Driving Forces tradition as a process-oriented perspective on SR in its own right. Research within this tradition examines how learners initiate, sustain, monitor, and regulate motivation, affect, and goal pursuit across learning situations (e.g., Miele and Scholer 2018, Hattie et al. 2020). From this perspective, SR is fundamentally energized and directed by motivational and affective states that fluctuate in response to contextual demands and perceived progress.

Substantial overlap nevertheless exists with the Learning Activities tradition because learners’ engagement in learning activities depends strongly on their current motivational and affective states. Consequently, many models centered on Learning Activities selectively incorporate constructs associated with Driving Forces, such as self-efficacy, achievement goals, interest, emotions, and motivational regulation (Boekaerts, 1996; Efklides & Schwartz, 2024; Pintrich & Zusho, 2002; Zimmerman, 2000). However, these integrations are typically conceptualized from the perspective of regulating learning activities rather than providing a comprehensive account of motivational and affective processes themselves.

At the same time, research within the Driving Forces tradition increasingly examines how motivational and affective processes interact with metacognitive monitoring, standards, and perceptions of progress during goal pursuit (Hattie et al., 2020; Miele & Scholer, 2018). Monitoring perceived progress toward standards can itself elicit affective reactions, which may subsequently influence attention, memory, metacognitive judgments, and ongoing regulatory behavior (Baumeister et al., 2015; Carver & Scheier, 1990, 1998; Fredrickson & Branigan, 2005; Levine & Burgess, 1997). These reciprocal interactions illustrate how motivational and affective dynamics may both shape and be shaped by ongoing regulatory activity across learning situations.

In essence, the *hard core* of the Driving Forces research tradition assumes that SR is an energized process initiated and sustained by motivational and affective states, which determine the direction and intensity of regulatory effort. Intrinsic motivation, for instance, may fuel engagement in Learning Activities by providing the persistence and effort required for sustained regulation. Conversely, successful engagement in Learning Activities may strengthen motivational states by fostering a sense of autonomy and competence, key components of self-determination theory (Ryan & Deci, 2000).

From a Lakatosian perspective, the *hard core* of this tradition identifies motivational and affective processes as the primary catalysts of regulatory behavior, whereas its *protective belt* increasingly overlaps with constructs associated with Learning Activities, Stable Dispositions, and, to a lesser extent, Limited Resources. Emerging integrative work has particularly emphasized the connection between Driving Forces and Learning Activities. For example, meta-analytic evidence demonstrates that motivational regulation strategies are systematically related to learners’ engagement in learning activities and academic achievement (Fong et al., 2024). Similarly, motivational constructs such as achievement goals, self-efficacy, and emotions are frequently integrated into models of metacognitive regulation and strategy use (Boekaerts & Niemivirta, 2000; Efklides, 2011; Schunk & Greene, 2018a; Zimmerman, 1986).

At the same time, these integrations remain selective and are often rooted in specific research perspectives. While motivational and affective processes have long been incorporated into models of Learning Activities (e.g., Winne and Hadwin 2008), a comprehensive integration of the broader range of Driving Forces with Stable Dispositions and Limited Resources remains comparatively underdeveloped. Nevertheless, both Learning Activities research and broader motivational research consistently demonstrate that these Driving Forces constitute a critical condition for self-regulation: without the energizing and directional functions provided by these processes, regulatory skills and strategies are unlikely to be enacted effectively.

### Learning Activities

A final cornerstone of research on SR in education concerns the active learning processes learners engage in while working on academic tasks. These learning processes are at the core of models of SRL (e.g., Panadero 2017, Puustinen and Pulkkinen 2001, Tinajero et al. 2024) and of research on learning strategies (Hattie & Donoghue, 2016). The roots of this tradition lie in research on metacognition, that is, the monitoring and regulation of cognitive processes (Flavell, 1979). Contemporary SRL models examine how learners regulate cognition, behavior, and metacognition during learning episodes across educational contexts (Pintrich, 2004; Winne & Perry, 2000; Zimmerman, 2000). Whereas some models emphasize the competencies, knowledge structures, and conditions required for effective regulation (Boekaerts, 1996; Efklides, 2011), process-oriented approaches focus on how Learning Activities unfold dynamically across phases of preparation, performance, and adaptation (Winne & Hadwin, 1998, 2008; Zimmerman, 2000).

While SRL models primarily focus on the regulation of learning activities, many selectively incorporate constructs associated with other research traditions. For instance, influential models proposed by Boekaerts (1996), Borkowski et al. (2000), Efklides (2011), Pintrich (2000), Winne and Hadwin (1998), and Zimmerman (1989) integrate constructs such as motivational processes, affect, cognitive resources, strategic competencies, or interactions between learner and task characteristics (Boekaerts, 1996; Efklides, 2011; Pintrich, 2000; Winne & Hadwin, 1998). However, these integrations are typically conceptualized from the perspective of regulating learning activities rather than providing comprehensive accounts of the broader constructs represented in traditions such as Driving Forces, Limited Resources, or Stable Dispositions. Consequently, this research tradition strongly emphasizes the contextual and dynamic nature of learning processes and increasingly calls for the investigation of contingent, context-sensitive patterns of regulation as they unfold over time (Ben-Eliyahu and Bernacki, 2015; Winne, 2020; see also Bernacki, 2025; Fan et al., 2022).

Whereas some traditions conceptualize SRL primarily as a stable or trait-like characteristic (e.g., measured as the typical frequency of strategy use; Hong and O’Neil, 2001), our focus on Learning Activities emphasizes SRL as a task-specific and situational process (Winne & Perry, 2000). Most SRL models conceptualize learning as a cyclical and recursive process involving phases of preparation, performance, and appraisal (Schunk & Greene, 2018a). During these phases, learners establish goals or standards, enact and adapt learning activities, monitor progress, evaluate outcomes, and adjust future approaches to learning (Boekaerts, 1996; Pintrich, 2000; Winne & Hadwin, 1998; Zimmerman, 2000). Across all phases, metacognitive monitoring and control are considered central mechanisms enabling the continuous adaptation of learning activities (Butler & Winne, 1995; Griffin et al., 2013; Winne, 2001). Importantly, several models conceptualize these processes as flexible and recursive rather than strictly linear (Butler & Winne, 1995; Carver & Scheier, 1990; Thillmann et al., 2009; Winne, 2001).

Research within this tradition increasingly relies on process-oriented methodologies, including self-reports, trace data, log files, eye tracking, and multimodal analytics, to investigate learners’ strategies, metacognitive monitoring, and regulatory adaptations during learning (Azevedo et al., 2018; Bannert et al., 2014; Scheiter & Gerjets, 2007). Although connections between fine-grained cognitive paradigms and broader educational SRL models remain somewhat limited (De Bruin & van Gog, 2012; Panadero, 2017; Schleinschok et al., 2017; Winne & Azevedo, 2014), recent advances in digital learning environments, learning analytics, and computational methodologies have enabled highly detailed real-time assessments of Learning Activities and SRL processes (Azevedo & Gašević, 2019; Bernacki, 2024, 2025; Giannakos et al., 2022; Winne, 2017, 2022). These developments promise increasingly fine-grained insights into the contextual and dynamic nature of self-regulatory learning processes.

In summary, the *hard core* of the Learning Activities research tradition assumes that SR is a dynamic, cyclical process of task-specific cognitive and metacognitive events (i.e., preparation, performance, and appraisal), where success depends on the strategic monitoring and active adjustment of behavior in response to situational demands. Research in this tradition provides a crucial lens for understanding the active, goal-directed, and cyclical nature of how learners engage with and regulate their own learning. Although it focuses primarily on cognitive and metacognitive processes through a pedagogical-psychological lens, researchers increasingly recognize that the effectiveness of these activities is intertwined with factors such as motivation, personal dispositions, and the control of cognitive resources.

From a Lakatosian perspective, whereas the *hard core* of this tradition focuses on the situational processes involved in regulating learning activities rather than on between-person differences, a more comprehensive understanding of SR emerges when its *protective belt* is connected with other traditions. Emerging integrative reviews illustrate increasing connections between Learning Activities and constructs associated with Driving Forces, Limited Resources, and Stable Dispositions. For example, an umbrella review of technology-supported learning research highlighted the growing incorporation of motivational processes, cognitive resources, and learner characteristics into models of learning activities and metacognitive regulation, although such integrations often remain selective and theoretically uneven (Prasse et al., 2024). Similarly, broader reviews have linked learning strategies and metacognitive regulation to motivational, cognitive, and dispositional factors associated with academic achievement (Richardson et al., 2012; Theobald et al., 2026).

For instance, a growing body of literature cuts across the boundaries of the Learning Activities and Limited Resources traditions by connecting metacognitive regulation with executive functions and cognitive load (e.g., Roebers 2017, Seufert 2018, Seufert 2020). These developments illustrate how the regulation of learning activities is both supported and constrained by the cognitive resources and dispositional characteristics that learners bring to a task.

## Integration – A framework for Self-Regulation in Education

The field of SR in education encompasses a wide array of theoretical approaches and empirical findings that, as noted above, are often rooted in distinct research traditions. Despite their varied emphases, these traditions can be analytically unified by identifying their core explanatory mechanisms, what Lakatos (1970; 1978) describes as their *hard cores*, and examining how their central constructs function in relation to one another. In this section, we propose an integrative framework that organizes these traditions around their foundational assumptions and explores how their constructs interact in educational contexts. The goal is not to introduce new mechanisms or causal explanations, but to embed existing models and findings conceptually within a broader architecture of SR. This integration serves two primary purposes: First, it provides a conceptual map that helps researchers situate existing models, thereby clarifying points of convergence and divergence. Second, it underscores the need for multi-level, context-sensitive investigations that account for interactions across motivational, cognitive, strategic, and dispositional systems.

Stable Dispositions offer a trait-based perspective on SR, highlighting how relatively enduring characteristics, such as conscientiousness, neuroticism, or grit, influence how learners typically engage with regulatory demands and academic tasks (Costa & McCrae, 1992; Duckworth et al., 2007). Although such traits may not themselves constitute self-regulatory processes, they critically determine the likelihood, quality, and consistency with which these processes are deployed across contexts.

Limited Resources, by contrast, refer to domain-general cognitive capacities such as working memory, attentional control, and inhibition, core mechanisms that form the architecture for regulatory functioning (Hasher & Zacks, 1988; Miyake et al., 2000). These capacities constrain what learners can monitor, maintain, or shift between in real time, making them central to effective SR, especially under cognitively demanding conditions. Unlike Stable Dispositions, cognitive resources are susceptible to intra-situational fluctuations (e.g., increased cognitive load due to task complexity or emotional interference).

Driving Forces capture the motivational and affective processes that energize or hinder learners’ engagement with learning tasks (Boekaerts, 1996; Efklides, 2011; Pintrich, 2000; Zimmerman, 2000). These forces include goals, values, emotional states, and volitional capacities, which shape the intensity and persistence of self-regulatory efforts. Within this tradition, SR is not only about managing cognition but also about sustaining motivation, managing affect, and maintaining engagement over time.

Learning Activities encompass research focused on the strategies learners use to regulate their cognition and behaviors during task engagement. Grounded in cognitive and metacognitive theories, this tradition examines the cyclical nature of planning, monitoring, and evaluating academic activities (Greene & Azevedo, 2009; Winne & Hadwin, 1998). These dynamic processes are highly task-specific and sensitive to contextual cues.

**Fig. 2:**
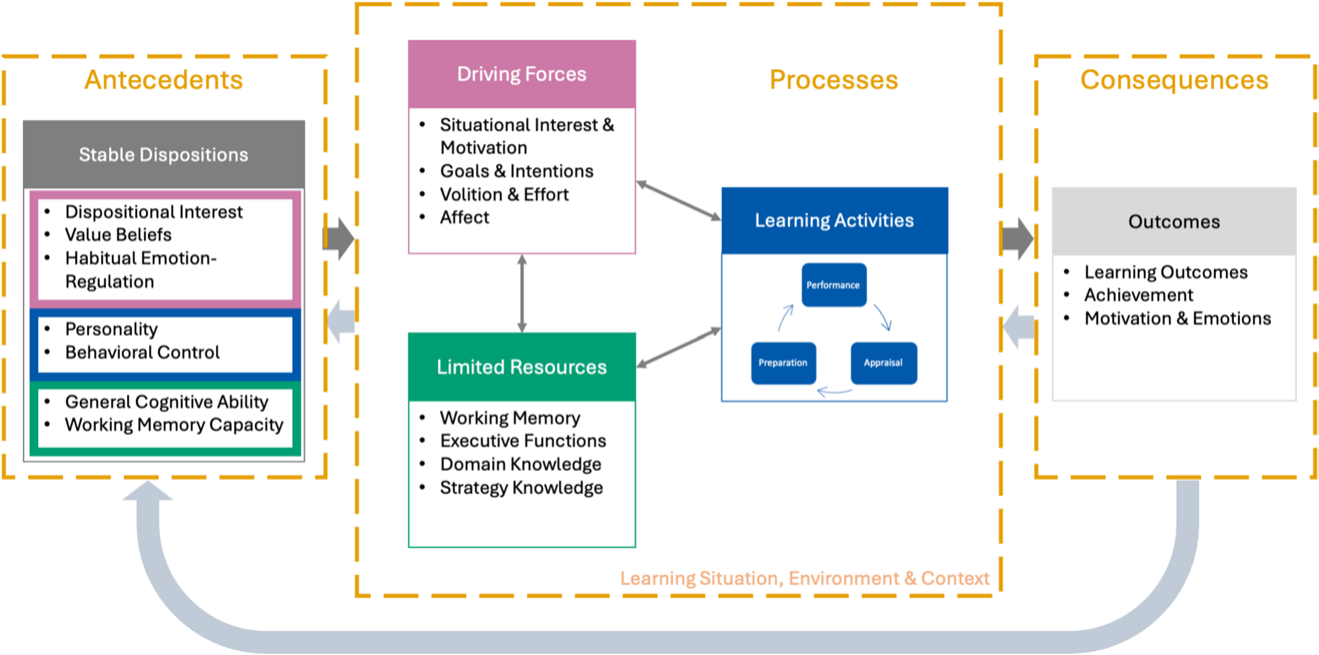
The integrative framework for SR in education. Note. Arrows indicate hypothesized relationships between framework components. Darker arrows represent temporal and causal influences, whereas lighter arrows denote reciprocal effects. The color-coded mappings between Stable Dispositions and other parts of the model illustrate primary associations. However, other direct and indirect influences exist between all components (e.g., the association between personality and Driving Forces), reflecting the deeply integrated nature of the SR system. The lists of concepts within the main framework components are exemplary rather than exhaustive

At its core, our integrative framework conceptualizes SR as a person-situation interaction, wherein stable learner characteristics shape how situation-specific self-regulatory processes unfold and influence educational outcomes (see Fig. 2). This view demands the integration of constructs that differ in granularity and temporal scope. For example, research on learning strategies ranges from real-time metacognitive judgments (Greene & Azevedo, 2007) to strategy use across academic semesters (Dunlosky & Thiede, 2013). Because the framework is grounded in the distinction between different explanatory mechanisms, some dispositional traits may encompass constructs more commonly associated with other traditions. For instance, working memory capacity and well-developed individual interest are Stable Dispositions, whereas their situational counterparts, working memory load and triggered situational interest, vary with task demands and context.

This leads to the second core tenet of the framework: a dynamic interplay among Driving Forces, Limited Resources, and Learning Activities, continually shaped by Stable Dispositions, unfolds within specific learning environments. These components jointly shape learning progress, ultimately contributing to educational and personal outcomes.

Although analytically distinct, these traditions are not mutually exclusive. Rather, their mechanisms frequently intersect in redundant, complementary, or compensatory ways. Redundancy arises when similar outcomes, such as persistence, are explained by different constructs (e.g., conscientiousness versus personal interest). Without theoretical integration, such overlap can lead to measurement redundancy and ambiguous interpretations (Credé et al., 2017).

More constructively, mechanisms often function complementarily. For instance, effective learning strategy use typically requires coordination among metacognitive monitoring (Learning Activities), motivational support (Driving Forces), and adequate cognitive resources (Limited Resources). A student’s ability to adapt strategies based on performance feedback depends on available working memory and is sustained by motivational goals such as a mastery orientation or self-efficacy (Ames, 1992; Bandura, 1986).

Whereas these examples demonstrate the complementary nature of SR processes, compensatory interactions among SR constructs are also expected. For example, a highly motivated student may compensate for limited EF by increasing time and effort. Conversely, strong cognitive resources may offset weaker motivation or strategy use. Similarly, a conscientious student may persist despite low situational interest, whereas another might rely on situational engagement to compensate for lower trait-level persistence (Trautwein et al., 2015). These examples illustrate why SR should be understood as a system of interdependent processes rather than as isolated mechanisms.

Crucially, these interactions are context dependent. Task complexity, domain specificity, and instructional supports modulate the relative importance of different regulatory components. High task demands can overload cognitive capacity, thereby impeding metacognitive monitoring (Paas & Sweller, 2012; Sweller, 2005). Emotionally charged tasks may hinder EF or motivation, requiring external scaffolds (D’Mello & Graesser, 2013; Kensinger & Corkin, 2003). Stable traits also express themselves differently across contexts. For example, neuroticism may have more pronounced effects in high-stress environments, while conscientiousness may buffer against distractions when structural supports are absent.

Over time, these person-context interactions shape learners’ longer-term development (Murayama, 2022; Wrzus & Roberts, 2017). Repeated success in SR fosters self-efficacy and a positive academic self-concept, whereas chronic difficulty may lead to disengagement or maladaptive beliefs. Thus, the framework provides a structure for examining how situated processes give rise to enduring traits, and how those traits in turn shape situational dynamics.

Finally, self-regulatory processes occur across multiple levels of granularity and timescales. Drawing from action regulation theory (Hacker, 2003), we propose a hierarchical model of nested self-regulatory cycles. These cycles can be conceptually segmented into three distinct levels: the macro-level (e.g., managing long-term goals such as preparing for an examination across multiple study sessions or an entire semester), the meso-level (e.g., executing within-session cycles of planning, performance, and appraisal), and the micro-level (e.g., adjusting strategies in response to real-time feedback or moment-to-moment monitoring).

Importantly, success or failure at one level can systematically influence processes at another: a poor result on a micro-level self-test may trigger motivational regulation inflected by trait conscientiousness, leading to the adoption of deeper strategies in future meso-level sessions. For instance, daily (meso-level) study satisfaction mediates the effect of SRL behaviors (Learning Activities) on a student’s intrinsic and extrinsic motivation (Driving Forces) the following day (Bellhäuser et al., 2021). This daily feedback loop highlights how the success of specific regulatory actions influences subsequent motivational states. Furthermore, such longitudinal tracking demonstrates the utility of macro-level distinctions, as these effects can vary significantly between the lecture period and the exam preparation period. Conversely, the overall process of examination preparation is itself embedded within broader academic goals or long-term study programs. While the framework accommodates this multilevel structure, integrating across levels poses significant methodological challenges, particularly when measurement scales or granularities differ (e.g., tracking transient cognitive load across an entire academic semester; see also Becker et al., 2024).

Nonetheless, such an approach is essential for capturing the full complexity of SR. Furthermore, we acknowledge that not all learning tasks require active self-regulation. Learners may forgo explicit regulatory activities through automatic processes (Norman & Shallice, 1986) or when a task can be resolved entirely through the deployment of prior knowledge without the need for strategic intervention (Borkowski et al., 2000). While such situations are critical for understanding the full spectrum of academic behavior, they represent the baseline of non-regulated performance. Our framework focuses specifically on the active regulatory system that is recruited when these automatic processes are insufficient to meet task demands. By isolating these mechanisms, we aim to provide a common conceptual metric for the instances where SR becomes the decisive factor in academic success.

## Discussion

In the remainder of this paper, we discuss four critical aspects of the broad framework for SR in educational contexts outlined above. First, we address how to resolve, on a theoretical level, the jingle-jangle fallacies that inevitably arise within an overarching framework that embraces diverse conceptualizations and constructs. Second, we examine how researchers might methodologically manage the diversity of constructs involved in the framework, such as by leveraging advanced statistical approaches and machine learning techniques to model multi-level influences. Third, we outline what empirical evidence demonstrates the importance of applying a comprehensive framework to SRL to better understand how SR dynamically unfolds across different learning situations. Finally, we sketch a comprehensive agenda for future research derived from this integrative architecture.

### Theoretical Implications of the Framework

We initiated this paper with the observation that research on SR in educational contexts appears largely fragmented due to distinct research traditions that have developed in relative isolation from one another. A primary source of this fragmentation stems from the jingle-jangle fallacies, wherein highly similar constructs based on overlapping indicators and core assumptions are labeled differently (the jangle fallacy), or distinct constructs involving different indicators are labeled identically (the jingle fallacy). Throughout this paper, we have provided an overview of constructs from diverse research traditions that examine SR in education from specific conceptual perspectives, utilizing a Lakatosian approach (1970; 1978) to delineate four distinct research traditions. Each tradition shares a specific type of explanatory construct or entity as its theoretical *hard core* (i.e., Stable Dispositions, Limited Resources, Driving Forces, and Learning Activities), around which we organized the representative constructs investigated within these explanatory paradigms.

An analysis of these traditions reveals prominent jingle-jangle fallacies in two distinct ways: first, new terms for constructs are introduced to highlight specific facets of a general phenomenon that are neglected by existing frameworks. Second, identical terminology is applied to fundamentally different constructs. For instance, within the domain of self-regulatory dispositions, the concept of grit emphasizes long-term persistence in goal pursuit to a greater degree than the broader trait of conscientiousness. Conversely, the concept of trait self-control focuses more narrowly on the capacity to override impulses, manage competing motivations, and resist distractions. However, it omits other structural dimensions of conscientiousness, such as orderliness, dutifulness, achievement striving, or deliberation, that do not directly involve volitional inhibition in a strict sense. Nevertheless, all three constructs rely on highly overlapping behavioral indicators and shared core assumptions.

Parallel to this dispositional example, each category of explanatory approaches contains research programs that display a substantial family resemblance in terms of overlapping core assumptions or indicators, alongside notable differences in specific assumptions stemming from the distinct areas of application for which the constructs were originally developed. Conversely, many instances exist where identical terms, such as inhibition, EF, or engagement, are used differently across competing conceptualizations. This latter issue, however, may be less severe than jangle fallacies, as these conceptual differences can often be resolved by specifying the exact subconstruct under investigation, such as by deploying more precise terminology (e.g., cold versus hot EF; cognitive versus emotional inhibition; or cognitive, affective, or behavioral engagement).

Based on the synthesized collections of constructs and the Lakatosian perspective adopted herein, we identify three distinct pathways to address jingle-jangle fallacies, particularly the jangle fallacy of introducing redundant construct terminology. These options comprise: (1) pursuing the traditional approach of identifying the single optimal construct, (2) establishing a problem-centered rather than a theory-centered research program in the sense of Herman (1979), or (3) adopting the structuralist view of theories formulated by Balzer et al. (1987) to differentiate between theories and concepts as formal structures on the one hand, and their respective sets of intended applications on the other, which defines the semantics of those formal structures.

1. Traditional approach: Many discussions in the literature focus on questions such as what the optimal definition or measurement of a concept would be, or which construct from a set of similar alternatives would prove most useful in a particular context. Based on this approach, jingle-jangle fallacies should eventually be resolved by determining empirically which of the overlapping constructs is superior overall (the jangle fallacy) or which of the competing definitions or measurements of a construct is most valid overall (the jingle fallacy). In our view, this type of definitive solution is rarely achieved because each overlapping construct and alternative definition typically possesses empirical evidence in its favor that its proponents can leverage to argue for its utility. In line with this reasoning, the number of distinct measures used in psychological research has continuously increased over recent decades (Anvari et al., 2025). Moreover, a distinct trend has emerged toward deploying highly specific measures tied to localized tasks rather than broader measures covering more abstract constructs (De Boeck et al., 2023). This shift naturally yields stronger predictions for the targeted tasks, albeit at the cost of lower conceptual abstraction. According to De Boeck et al. (2023), psychological constructs can be conceptualized as inhomogeneous spaces that are covered by different measures only to a certain extent, potentially even without creating overlapping areas between those measures. From this perspective, it may be advantageous to combine complementary measures into a unified compound metric that more comprehensively captures the overarching theoretical construct.
2. Problem-centered research programs: Herman (1979) advanced the proposition that psychological science does not exclusively comprise the singular type of research program postulated by Lakatos (1978). Research programs according to Lakatos consist of a theoretical *hard core* of foundational assertions, which is protected from immediate falsification by a *protective belt* of auxiliary hypotheses, methods, heuristics, and operational constructs. This belt is deployed to account for anomalous evidence that threatens the *hard core* and to guide the ongoing development of the research program. Herman (1979) argues that psychology encompasses not only theoretical research programs but also technological research programs that anchor a specific practical problem as their *hard core*. Within these technological programs, theoretical assumptions are not part of the immutable *hard core* but instead function within the more malleable and flexible auxiliary belt. From this perspective, alternative overlapping constructs alongside competing definitions and measurements can be viewed as a dynamic toolbox within a problem-centered research program dedicated to evaluating and optimizing learners’ self-regulatory capabilities. Interestingly, Peirce (1997) articulated closely related principles regarding how scientific inquiry should be organized around concrete problems. His pragmatist philosophy emphasized that genuine philosophical and scientific inquiry emerges from actual doubts and practical challenges rather than from artificial or purely academic concerns. From the perspective of a problem-centered research program, researchers might look past a lack of theoretical consistency across constructs and measures and, instead, appreciate the diversity and flexibility of the conceptual tools available to address the focal problem.
3. Structuralist view of theories: This formal approach to scientific architectures extends several core dimensions of Lakatos’ philosophy of science and has been operationalized in detail by Balzer et al. (1987). Within this framework, research programs are characterized as formal theory nets consisting of a *hard core* (the basic theory element) and supplementary, highly specialized theory elements that embed auxiliary assumptions tailored to specific domains of application (e.g., localized tasks, contexts, or measurement modalities). The foundational premise of this approach is to disentangle the formal structural equations of a theory net from the semantics of its constituent constructs. The meaning of a given construct is jointly determined by: (1) its formal, mathematical relations to other constructs within the network, and (2) the specific set of intended applications designated for the theory elements that operationalize that construct. These configurations of specialized theory elements and intended applications are not statically predefined. They rather evolve dynamically over time, guided by Lakatosian heuristics. Consequently, the structuralist perspective eliminates semantic disputes over terminology, nomenclature, and superficial verbal definitions (for an application of this perspective to motivational constructs, see Murayama et al. 2019, Murayama and Jach 2025). Instead, researchers mathematically define the formal properties of a construct *X* and systematically characterize the explicit set of situations *S* to which it is intended to apply. Given that the jingle-jangle fallacy is fundamentally a dispute over linguistic labels and nominal definitions, reconstructing these conflated cases as structuralist theory nets offers a rigorous, viable resolution (for a precedent disentangling motivational constructs within the Rubicon model of action phases and the cognitive architecture ACT-R, see Gerjets 1997).

To ground these philosophical options within current educational literature, we can analyze the construct overlap among conscientiousness, grit, and trait self-control as a classic jangle fallacy. Under the traditional paradigm (1), researchers would debate which of these three constructs is most psychometrically optimal and should consequently dominate research and educational practice. Alternatively, under a problem-centered paradigm (2), we can assume that although these constructs emerge from distinct theoretical traditions, they function as interdependent components of a unified, problem-centered research program that targets a shared practical challenge: namely, managing students’ behavioral SR in educational contexts. Within this framework, the diverse constructs and their corresponding instruments cease to operate as theoretical competitors. Instead, they constitute a complementary toolbox whose individual tools are differentially suited to address distinct facets of the focal problem. Finally, under the structuralist paradigm (3), researchers can integrate all three constructs into a cohesive theory net anchored by a unifying basic theory element that formally expresses their shared core assumptions. Branching from this foundational node, specialized theory elements can be tailored to each of the three individual accounts, with each node paired with its own precise set of intended applications, such as situations requiring long-term, multi-semester persistence versus situations involving immediate volitional temptation. This architecture implies that while one account may be optimally suited for describing a specific situational profile, an alternative account may prove superior for another. Because they are integrated within a single structural net, these models maintain a complementary rather than an adversarial relationship, wherein the ultimate empirical success of each specialized branch across its designated applications dictates its functional scope and utility.

This strategy of reconstructing hierarchical theory nets with explicit intertheoretical relations offers a systematic methodology to clarify the messy conceptual overlaps among terms often used interchangeably in educational research, such as SRL, EF, inhibition, self-control, and delay of gratification. This structuralist reconstruction would yield a basic SR theory element capturing the foundational assumptions shared across all these constructs. Branching from this, a specialized theory element for SRL would formalize the specific monitoring and control of cognitive and metacognitive activities and strategies within its designated set of intended applications: namely, formal and informal learning situations.

Concurrently, a cognitive control theory element would formalize assumptions regarding competing goals and impulses, mapped to its own explicit set of intended applications involving immediate temptations. The delay of gratification construct would then function as a further specialization of this cognitive control node, explicitly calibrated for situations where an immediate reward threatens a more substantial, distal outcome. To complete the network, the general EF theory element and its specific sub-element, inhibition, can intersect with the cognitive control node to specify the low-level, bottom-up cognitive mechanisms that support resistance against immediate gratifications. Crucially, because it is a network rather than a rigid tree, the EF element can simultaneously cross-connect with higher-level theory elements like SRL to operationalize the top-down cognitive processes required to plan, implement, and evaluate complex learning strategies.

### Methodological Implications of the Framework

The integrative framework presented herein conceptualizes SR as a complex, dynamic person-by-situation system that unfolds across multiple levels of analysis. Consequently, this architecture raises critical methodological considerations for future research. Accurately capturing the dynamic interplay among stable dispositions, transient motivational states, cognitive resources, and strategic behaviors, both within and across shifting learning contexts, requires empirical approaches that are equally multi-layered and flexible.

A major empirical challenge lies in integrating variables across disparate levels of granularity and temporal scopes. As outlined in our framework, self-regulatory processes operate hierarchically, spanning from micro-level mechanisms within a single task (e.g., metacognitive judgments or shifts in attention) to broader cycles of regulation across distinct learning sessions, semesters, or entire academic careers. Each of these analytical levels introduces unique measurement demands, and these demands are rarely aligned. For example, fine-grained indicators of working memory load or momentary motivation are typically assessed within narrowly defined, time-limited tasks, whereas traits such as conscientiousness or habitual strategy use are captured across much broader timescales. This integration challenge is further compounded by a well-documented trade-off between specificity and generalizability. Process-oriented research programs, particularly those focused on Learning Activities or Limited Resources, frequently leverage high-frequency stream data (e.g., eye-tracking, digital log files, or physiological monitoring) to draw precise inferences about regulatory processes within localized learning environments (e.g., Appel et al. 2019, Azevedo et al. 2010). While these approaches yield deep insights into situational dynamics, they often lack external validity beyond the specific task or context under investigation.

In contrast, models grounded in abstract traits or generalized strategies tend to yield broader predictive validity across contexts, yet they offer less insight into moment-to-moment regulatory behaviors and direct causal mechanisms. Moreover, constructs from divergent traditions are often operationalized via fundamentally different instruments, ranging from self-report surveys and cognitive tasks to behavioral trace data. As integrative research has shown, these methodological fractures can systematically obscure theoretically expected relationships. For instance, Eisenberg et al. (2019) demonstrated that survey measures of SR showed weak statistical correspondence with behavioral and cognitive task data, even when both sets of variables ostensibly reflected related latent constructs. Conversely, recent comparable investigations of curiosity found meaningful associations across different measurement modalities, but highlighted that psychometric surveys and behavioral task performance capture distinct facets of the construct, a nuance that warrants caution when comparing results across disciplinary boundaries (Jach et al., 2024). Integrative frameworks addressing SR in education must systematically account for these methodological discrepancies as well.

A particularly persistent barrier to advancing self-regulatory research in education is the structural measurement gap separating retrospective self-reports from the dynamic, real-time regulatory processes they are intended to capture (Arizmendi et al., 2023; Baker et al., 2020; Du et al., 2023; Roth et al., 2016). Our framework highlights a complex state-trait mapping of constructs across traditions, such as when habitual emotion regulation capacities actively shape affect within a localized learning situation. Yet, the retrospective self-reports traditionally used to assess habitual emotion regulation capture an aggregated, stable trait, whereas the momentary affect being regulated is a highly fluid state variable typically captured via fine-grained, in situ methods such as experience sampling methodologies. Consequently, an acute mismatch in granularity arises when these distinct levels of analysis are treated interchangeably. This granularity gap is further compounded by a corresponding semantic gap. The uncoordinated proliferation of psychological measures has led to widespread jingle-jangle fallacies, wherein distinct terms mask identical underlying constructs or identical terms obscure fundamentally different psychological processes (Anvari et al., 2025; Elson et al., 2023).

Collectively, these measurement and conceptual inconsistencies stifle empirical progress by obstructing the establishment of common metrics across distinct research traditions. While the field increasingly seeks alternatives to traditional questionnaires, process-oriented measures, such as digital log data, present their own distinct challenges as high-inference data sources. Specifically, latent constructs are frequently mapped onto situation-specific behavioral traces (e.g., interpreting prolonged reading times within a digital learning environment as deeper cognitive processing) that remain inherently ambiguous without contextual grounding. For instance, identical behavioral stretches could alternatively reflect disengagement, distraction, or a profound lack of comprehension. This diagnostic ambiguity introduces a critical bottleneck for advanced analytical methodologies: supervised machine learning models lack reliable ground-truth labels if retrospective data are too granularly coarse and process data prove too behaviorally ambiguous. To bridge this methodological fracture, researchers must transition toward multimodal, in situ assessment designs. Techniques such as experience sampling methodologies and situated thought probes offer a promising resolution: they preserve the temporal specificity of behavioral trace data while simultaneously providing the immediate semantic context required to validate high-inference sensor or log data, thereby generating the reliable criteria necessary for training robust predictive models and achieving meaningful theoretical integration.

Our framework suggests, in alignment with recent meta-analyses addressing the multimethod assessment of SRL (e.g., Bühler et al. 2025, Cleary et al. 2023, Ruhl et al. 2025), that a productive path forward lies not in choosing between these disparate approaches, but in designing frameworks to empirically link and align them. This alignment necessitates an expanded methodological toolkit capable of moving beyond traditional, hypothesis-based statistical procedures. To address this empirical frontier, we propose two complementary avenues for methodological innovation. First, computational models can provide a mechanistic explanation for how higher-level, macro-phenomena emerge from basic, lower-level components. For example, Murayama and Jach (2025) demonstrated how formalizing localized computational mechanisms can naturally give rise to broader psychological phenomena traditionally classified as “motivation” or “needs,” thereby offering a trajectory for theory development that does not solely rely on inductive empirical data. Within this paradigm, the analytical focus shifts toward validating a formal theory at a micro-level, exploring how its localized algorithmic mechanisms generate a higher-level construct as a systematic emergent property.

Second, to address the empirical challenge of integrating numerous and frequently overlapping predictors, machine learning methodologies offer a highly promising alternative. In contrast to classical statistical approaches designed primarily for hypothesis-driven theory testing, machine learning techniques are uniquely suited to the high-dimensional, multimodal nature of self-regulatory data (Yarkoni & Westfall, 2017). They possess the capacity to model highly heterogeneous data streams, ranging from stable dispositional traits to sensor-based process measures, thereby allowing for the formal integration of variables across divergent research traditions and levels of granularity. Crucially, machine learning can be leveraged to develop unobtrusive, multimodal process measures based on digital sensors such as touch surfaces, eye trackers, computer-vision cameras, and physiological recordings to extract indicators related to moment-to-moment fluctuations in Driving Forces (e.g., measuring engagement; Goldberg et al. 2021). This approach can similarly detect transient lapses in EF such as mind-wandering (Bühler et al., 2024a, b), capture real-time working memory load (Appel et al., 2021; Sevcenko et al., 2023), or classify active processing strategies (Kasneci et al., 2022). Continuous tracking is critical because capturing the situational unfolding of dynamic SR requires the simultaneous measurement of divergent motivational forces, resource investments, and learning activities as continuous, non-stationary processes rather than as static states.

Furthermore, machine learning algorithms, such as regularized regression, tree-based ensemble models, or deep neural networks, can accommodate complex non-linear interactions and uncover latent behavioral patterns without requiring the researcher to pre-specify rigid functional forms. This flexibility is particularly valuable for modeling the interdependent and compensatory dynamics described in our framework, such as when a learner’s high motivation directly compensates for acutely limited cognitive resources. The capacity of these algorithms to perform data-driven feature selection is also indispensable when researchers are confronted with a large array of conceptually close, yet distinct, constructs and measures. Importantly, these methodologies are equipped with robust, mathematically grounded mechanisms to mitigate overfitting and restricted generalizability, which remain persistent vulnerabilities in psychological science (e.g., Eklund et al. 2016). Regularization techniques, cross-validation, and dimensionality reduction systematically improve the robustness of performance estimates, ensuring that empirical findings generalize beyond the localized sample or specific task architecture. Finally, the application of explainable artificial intelligence (XAI) techniques (e.g., Lavelle-Hill et al. 2025) to these models can help unpack the specific structural weights of different variables, offering a robust empirical bridge between high-accuracy predictive performance and substantive theoretical interpretation.

Despite these promising developments, significant empirical challenges remain. Chief among these is the imperative to align distinct units of analysis across disparate data sources and temporal scales. Furthermore, while machine learning architectures provide invaluable insights into high-dimensional patterns of association, it is critical to recognize that standard predictive algorithms remain fundamentally correlational in nature. Interpretations of these models must therefore be treated with appropriate caution and, where feasible, be systematically triangulated with confirmatory or experimental research designs. Nevertheless, recent advances in causal machine learning, such as causal forests and orthogonal random forests, provide a sophisticated methodological avenue for isolating cause-and-effect relationships from complex, observational data structures (e.g., Oprescu et al. 2019, Rehill 2025). Ultimately, this integrative framework for SR in education demands an equally unified empirical toolkit capable of bridging micro- and macro-level processes, fleeting situational dynamics and stable dispositional influences, and divergent theoretical traditions. Methodological innovation, particularly through the symbiotic deployment of advanced machine learning and generative computational modeling, represents an indispensable pathway for testing non-linear hypotheses and cultivating comprehensive, ecologically valid models of SR within authentic educational environments.

Ultimately, to fully capitalize on the methodological opportunities outlined above, we advocate for multimodal, comprehensive data collection efforts designed to elucidate the empirical and conceptual relationships among divergent self-regulatory constructs. Open Science initiatives serve as an instrumental catalyst for this broader perspective, directly mitigating several systemic challenges in SR research through institutionalized practices such as study preregistration (Nosek et al., 2018), data and material transparency, and the systematic replication of existing findings (Obels et al., 2020). By emphasizing a rigorous focus on refined, fewer, and structurally stronger theories (Gehlbach & Robinson, 2021), these practices naturally align with our objective of synthesizing established research traditions rather than introducing additional niche constructs. This objective is directly coupled with ongoing efforts to consolidate psychological measures to counteract the uncoordinated proliferation of constructs driving current jingle-jangle fallacies (Anvari et al., 2025; Elson et al., 2023).

However, comprehensive, multi-method investigations of this scale are exceptionally costly to design, plan, and execute. To justify such resource-intensive endeavors, it is essential that the resulting datasets and underlying materials are shared openly, allowing the broader scientific community to exploit their full potential to resolve pervasive conceptual ambiguities and accelerate scientific progress (Colavizza et al., 2024). This open availability is particularly critical because isolated open-science efforts, such as sharing raw data files without the accompanying measurement instruments and documentation, can inadvertently amplify jingle-jangle fallacies (Peters & Crutzen, 2024). Furthermore, construct validity is frequently neglected even within formal, large-scale replication attempts (Flake et al., 2022). Thus, large-scale, encompassing studies must actively build upon the structural lessons learned from the replication crisis to establish a more robust psychometric foundation for the field. To operationalize this, we recommend conjointly investigating the four research traditions outlined in our framework through large-scale, multi-laboratory collaborative studies (Kvarven et al., 2020). Such initiatives would generate high-quality, universally accessible datasets that empower researchers to map the complex conceptual topology of SR in education, ultimately rendering future replication attempts more seamless, more logistically attractive, and more theoretically informative.

### Empirical Implications of the Framework

Due to the overarching nature of the proposed framework, comprehensive empirical validation remains a challenge, as the vast majority of existing literature has historically focused on only a narrow slice of self-regulatory research traditions. This theoretical fragmentation is reflected in the broader empirical landscape, where integrative efforts most commonly combine two research programs, whereas studies spanning three or all four traditions remain comparatively rare (see Table 1). To provide a focused overview within the constraints of this work, we primarily synthesized recent reviews and meta-analyses that explicitly integrate constructs across distinct traditions. Notably, with the sole exception of the comprehensive meta-analysis by Richardson et al. (2012), most integrative contributions have emerged only within the last few years, highlighting a rapidly growing recognition of the imperative for more comprehensive accounts of SR in education.

Existing integrative work has predominantly focused on pairwise combinations of Learning Activities with either Limited Resources or Driving Forces. For example, a recent review by Dörrenbächer-Ulrich and Bregulla (2024) synthesized research linking executive functions with SRL processes, thereby connecting the Limited Resources and Learning Activities traditions. Similarly, Fong et al. (2024) demonstrated in a comprehensive meta-analysis that motivational regulation strategies are intimately intertwined with learners’ active regulation of Learning Activities, highlighting the close interaction between Driving Forces and Learning Activities. More comprehensive approaches integrating three or more traditions remain comparatively scarce. One notable exception is the seminal meta-analysis by Richardson et al. (2012), which jointly examined cognitive abilities, personality traits, motivational constructs, and learning strategies as concurrent predictors of academic achievement.

Concurrently, existing integrative studies illustrate the structural limitations of the current literature. For instance, in their umbrella review on SRL within technology-enhanced learning environments, Prasse et al. (2024) noted that explicit connections between Learning Activities and Driving Forces remain relatively underdeveloped. Motivational constructs are frequently operationalized only as auxiliary variables adjacent to SRL processes, rather than treated as endogenous, core components of an overarching self-regulatory system, such as when motivation is categorized broadly under “human factors” (see Wong et al. 2019). Thus, while the growing body of literature bridging multiple research traditions provides indirect empirical support for our framework, it simultaneously underscores the critical need for systematic, theory-guided designs aimed at integrating self-regulatory constructs across divergent educational contexts, levels of analysis, and temporal scales.

**Table 1:** Illustrative examples of reviews integrating multiple research traditions in research on self-regulation in education

Study	SD	LR	DF	LA	Level of Analysis	Approach
Dörrenbächer-Ulrich and Bregulla (2024)		X	O	X	Cognitive process / SRL process	Review linking executive functions with SRL processes
Fong et al. (2024)			X	X	Situational regulation strategies	Meta-analysis of motivational regulation strategies in learning
Lavrijsen et al. (2022)	X	(X)	X		Trait and longitudinal achievement	Longitudinal relations between personality, motivation, and achievement
Nauzeer and Jaunky (2021)	X		X	(X)	Trait-level academic predictors	Meta-analysis of personality, motivation, and academic performance
Prasse et al. (2024)	O	(X)	X	X	Technology-supported SRL processes	Umbrella review of SRL support in technology-enhanced learning
Pretorius and Heyns (2026)	O	X	X	X	Executive functions and academic functioning	Scoping review of executive functions and academic performance
Richardson et al. (2012)	X	X	X	X	Multi-level academic predictors	Meta-analysis integrating cognitive, motivational, personality, and SRL predictors
Theobald et al. (2026)	O	X	X	X	Hierarchical SR architecture	Integrative review proposing a model linking EF, metacognition, SR, and SRL

Note. *SD* stable dispositions, *LR* limited resources, *DF* driving forces, *LA* learning activities, *X* explicit empirical integration or operationalization of the research tradition, *(X)* partial or indirect empirical operationalization, *O* conceptual or theoretical discussion without direct empirical assessment

A major reason for the fragmentation of research on SR in education lies in the extensive data collection required to assess multiple facets of the learner simultaneously. Integrating constructs across traditions often necessitates combining psychometric assessments of Stable Dispositions, cognitive tasks measuring Limited Resources, situational assessments of motivational and affective states, and process-oriented indicators of Learning Activities. Consequently, only few studies have attempted broader empirical integrations across self-regulatory components.

These systemic challenges of conceptual fragmentation and measurement misalignment are directly mirrored within the broader psychological sciences. For example, Eisenberg et al. (2019) conducted a large-scale ontological investigation of SR across behavioral task performance and self-report survey measures. Despite targeting ostensibly equivalent psychological constructs, cognitive tasks, frequently associated with the Limited Resources tradition, and survey instruments, commonly aligned with Stable Dispositions or Driving Forces, demonstrated surprisingly weak empirical overlap. Concurrently, however, that investigation demonstrated that data-driven, unsupervised machine learning approaches can uncover meaningful latent structures within and across distinct measurement modalities, thereby providing a robust empirical foundation for cross-tradition theoretical integration.

Collectively, existing evidence underscores that SR in education cannot be comprehensively captured through isolated lenses of motivation, cognition, stable dispositions, or localized learning strategies alone. Rather, educational outcomes emerge from dynamic interactions among these multi-layered components, unfolding heterogeneously across shifting contexts, distinct tasks, and divergent temporal scales. However, empirical research structurally capable of capturing these complex interactions remains scarce. In particular, a substantial portion of existing studies continues to rely heavily on retrospective self-reports, lacking the fine-grained process data required to trace self-regulatory dynamics in situ. Future research deploying multimodal assessment designs, intensive longitudinal frameworks, and context-sensitive analytical approaches is therefore imperative to empirically substantiate the non-linear interplay among the four research traditions outlined in the present framework.

## A Future Research Agenda Based on the Proposed Framework

What type of research on SR in education is needed in the future based on the theoretical, methodological, and empirical implications of the proposed framework? How can we derive hypotheses from the model? How can we argue in favor or against such an overarching model? How is it helping us to further academic research? We will focus on recommending seven topics that should be considered important parts of a future research agenda on SR according to our approach.

### Conduct More Comprehensive Studies That Embrace Broad Sets of Constructs

Investigating SR in a manner that synthesizes core assumptions from all four research traditions across disparate levels of granularity requires future designs to systematically transcend historical theoretical and methodological borders. As preliminary work by Wortha (2021) illustrates, embedding diverse methodological approaches within a single, unified sample allows researchers to evaluate overlapping and complementary self-regulatory constructs simultaneously. Such integrative frameworks are essential to clarify structural relations across divergent research traditions, ultimately fostering greater conceptual coherence and collaborative precision in interdisciplinary research on SR within educational settings.

To cover constructs from all theoretical research traditions introduced above requires the use of advanced assessment technologies to obtain not only (a) (self-)report data on personality traits, motivation, attitudes, beliefs, strategy use, metacognition, emotion regulation, and SR in realistic contexts (classrooms, homework). Rather, (b) performance data on mental abilities (e.g., working memory, EF, or emotion regulation) and on learning outcomes and educational attainment in different domains will be required, as well as (c) behavioral process data during learning in the lab and in the classroom (e.g., log files, ambulatory assessments, automated analyses of learner behavior). Moreover, (d) physiological data such as eye-tracking data (pupillometry, scan-path analysis) or data on electrodermal activity and heart-rate variability can be used as fine-grained measures of perceptual processing strategies, mental workload, and stress, as well as emotional reactions underlying learning or task performance.

### Use Comprehensive Data Sets to Derive New Testable Hypotheses

We want to state very clearly that the primary role of these data would not necessarily be to test specific hypotheses that have been derived from the model beforehand. Rather, its primary value might be that it allows us to develop new specific hypotheses based on exploratory analyses. It could be used to pose and answer numerous kinds of questions about relations between similar (i.e., same research tradition) or dissimilar (i.e., different research tradition) constructs and measures, and to evaluate the role of these constructs and relations for predicting learning outcomes. Coming up with these questions and having data at one’s disposal to answer them seems to be a good and empirically informed way, from our perspective, to derive new hypotheses that can be tested in subsequent, more specific studies that assess only the constructs required for hypothesis testing. Although the envisioned comprehensive studies could provide tentative evidence in favor of (or against) the overall structure of our model, it will surely not be possible to falsify or reject such a broad and underspecified model based on empirical data alone. Regarding this issue, we consider the model to be a basic conceptual structure that has not been developed to be corroborated or to be falsified as a whole, but that is intended as a framework that can be useful to sort constructs taxonomically, to unify research on SR in education, and to inspire the development of novel hypotheses. Essentially, we consider the model to function quite similarly to a Lakatosian *hard core*, i.e., as a set of foundational assumptions that are useful to organize a network of more specific and testable auxiliary hypotheses, methodological assumptions, and operationalizations. Therefore, although the model might be too overarching to allow for the derivation of specific, novel, and testable hypotheses by itself, we nevertheless see substantial added value in the model regarding its potential to further academic research.

### Develop Refined Methodologies for Construct Differentiation

Using data from comprehensive studies in a meaningful way will not only require advanced assessment technologies but also advanced statistical modeling techniques ranging from structural equation modeling and generalized additive models to machine learning. One focus should be on developing automated analyses of SR patterns using learning analytics, neural networks, support-vector machines, and other state-of-the-art machine learning techniques. These should be applied to the multiple streams of self-report, performance, process, and physiological data that should be recorded during the proposed comprehensive studies. SR patterns detected in physiological and behavioral process data can be connected to learning outcomes and performance data, as well as to (self-)report data, to better understand how complementary constructs work together in enabling successful SRL and how overlapping constructs might be better differentiated in terms of identifying which of their sets of intended applications, according to the structuralist view of theories, are actually successful applications. In this context, it will also be important to work on systematic literature reviews about groups of overlapping measures to better cover what is already known about their conceptual and empirical relations. This theoretical work is an important counterpart to the application of refined methodologies for construct differentiation in comprehensive studies.

### Explore Specific Compensatory Relationships Across Explanatory Approaches

Comprehensive studies and rich multimodal datasets can be used to better explore the multiple plausible candidates for compensatory relations between constructs from different research traditions. For instance, in the CONIC model (Song et al., 2020), a compensatory relationship between conscientiousness and interest regarding their effect on effort investment is emphasized. In conditions with high interest, learners low in conscientiousness are also assumed to invest effort (unlike in conditions with low interest). Thus, interest can compensate for a lack of conscientiousness, and conscientiousness can compensate for a lack of interest. Finding more cases of such systematic interdependencies between constructs from different research traditions would deepen our understanding of SR substantially and would even allow us to develop concepts for hybrid or adaptive interventions to support SRL. Potentially, methods from explainable artificial intelligence (XAI) can be used to inspect individual prediction models for each person in the dataset (e.g., Pereira et al. 2021), thereby allowing for the detection of even more complex compensatory effects at the individual level.

### Develop Hybrid and Adaptive Interventions to Support SR

There are numerous studies out there showing that SR interventions in educational contexts are useful (for an overview, see Dignath and Büttner 2008, Theobald 2021, Xu et al. 2023). However, insights like the ones envisioned above would allow for coming up with novel types of more specific interventions. For instance, hybrid interventions would allow for a concerted impact on different granularity levels by combining different interventions based on constructs from different research traditions. In this regard, cognitive or educational interventions addressing strategic or motivational aspects of SR can be combined with innovative neuro-technologies that target mental resources underlying self-regulation, such as EF or working memory. As an example, neurofeedback training for attention control shows a strong potential to be combined with educational interventions and has already been applied in virtual classrooms (e.g., Hudak et al. 2017), as well as during in-school attention training for attention-deficit/hyperactivity disorder (ADHD; Steiner et al. 2014). As another example, transcranial direct current stimulation (tDCS) has been shown to strongly improve the effects of working-memory training (Ruf et al., 2017).

Beyond these hybrid interventions, we might also come up with more adaptive interventions based on the proposed framework: The four theoretical research traditions regarding SR in education point to different problem areas that might be addressed by specific interventions, namely, supporting poor cognitive and metacognitive strategy use, improving weak motivation, compensating for a lack of mental resources, or coping with unfavorable personal dispositions. It is quite plausible that different learners struggle with different aspects of the SRL process and therefore need more adaptive interventions targeted to their specific problems. Based on meta-analytic evidence on differential effects of different SR interventions (e.g., Dignath and Büttner 2008, Theobald 2021, Xu et al. 2023), custom-tailored interventions could be developed and tested in subsamples of learners depending on their specific profiles of SR problems. Thus, our overarching conceptual framework can serve not only to inspire new and testable empirical hypotheses but also to come up with novel and integrated ideas for adaptive interventions.

### Conduct Longitudinal Studies to Investigate Causal Relationships Between Constructs

A final important part of a future research agenda on SR in education according to our approach would be to conduct larger longitudinal studies to better understand how constructs from different research traditions and at different levels of granularity causally impact each other over time. It would be highly valuable to develop a multi-cohort longitudinal panel study design that would allow researchers to study the development of SR in education from age 10 to age 20. Although SR is important in elementary schools as well, the emphasis on SR tends to increase greatly after the transition to secondary school, making this an ideal starting point for this type of investigation. The age range from 10 to 20 covers critical developmental years regarding educational decisions, personal and social development (including adolescence), and brain maturation. A study like this can serve not only to test specific theoretical hypotheses (using predefined subsets of the data) but also to explore causal interrelations of different SR measures in a comprehensive manner. The questions of how these measures interact with each other and how they influence learning processes and outcomes over the course of development are of particular interest. Such a longitudinal perspective would drastically improve the ability to gain insights into the interrelationships of constructs from different research traditions as compared to cross-sectional data.

Therefore, our call to action for the broader community interested in SR in education is to cooperate strongly to realize the comprehensive, longitudinal studies with the multi-perspective approach outlined above, finally achieving a better-grounded and more coherent understanding of the processes underlying SR in education.

### Outlook

Advancing and disentangling the complex landscape of SR in education requires a shift toward fundamentally integrative theoretical and empirical efforts. As our framework suggests, the path forward does not necessarily require the pruning of existing measures or the abandonment of established traditions. Instead, maintaining a sufficient rate of scientific progress depends on the strategic integration and refocusing of these research programs. By acknowledging the distinct *hard cores* of different traditions and aligning their auxiliary hypotheses, we can move away from fragmented, siloed research and toward a cumulative body of knowledge that accounts for both stable traits and dynamic processes.

This sharpened focus has implications far beyond the laboratory or the digital learning environment. A more coherent understanding of the mechanisms underlying SR provides a robust foundation for outreach and practical application. When researchers can clearly articulate how different regulatory components, from EF to motivational Driving Forces, interact within specific contexts, they can provide practitioners, educators, and policymakers with more targeted and effective interventions. Ultimately, by bridging the measurement and conceptual gaps identified here, we can ensure that the proliferation of research on SR translates into meaningful improvements in educational outcomes and learner agency.

## Data Availability

Not applicable
